# Multipotent mesenchymal stromal/stem cell-based therapies for acute respiratory distress syndrome: current progress, challenges, and future frontiers

**DOI:** 10.1590/1414-431X2024e13219

**Published:** 2024-10-14

**Authors:** M. Sababathy, G. Ramanathan, S. Ganesan, S. Sababathy, A.R. Yasmin, R. Ramasamy, J.B. Foo, Q.H. Looi, S.H. Nur-Fazila

**Affiliations:** 1 Department of Veterinary Pathology and Microbiology, Faculty of Veterinary Medicine, University Putra Malaysia, Serdang, Selangor, Malaysia; 2 Faculty of Computer Science and Information Technology, University Malaya, Kuala Lumpur, Malaysia; 3 School of Pharmacy, Management and Science University, Shah Alam, Selangor, Malaysia; 4 Faculty of Medicine and Defence Health, National Defence University of Malaysia, Sungai Besi, Kuala Lumpur, Malaysia; 5 Department of Veterinary Laboratory Diagnostics, Faculty of Veterinary Medicine, University Putra Malaysia, Serdang, Selangor, Malaysia; 6 Laboratory of Vaccines and Biomolecules, Institute of Bioscience, University Putra Malaysia, Serdang, Selangor, Malaysia; 7 Department of Pathology, Faculty of Medicine and Health Sciences, University Putra Malaysia, Serdang, Selangor, Malaysia; 8 Center for Drug Discovery and Molecular Pharmacology (CDDMP), Faculty of Health and Medical Sciences, Taylor's University, Subang Jaya, Selangor, Malaysia; 9 School of Pharmacy, Faculty of Health and Medical Sciences, Taylor's University, Subang Jaya, Selangor, Malaysia; 10 My Cytohealth Sdn. Bhd., Bandar Seri Petaling, Kuala Lumpur, Malaysia

**Keywords:** Multipotent mesenchymal stromal/stem cells, Acute respiratory distress syndrome, Treatment strategy, Gene modification, Preconditioning, Tissue engineering

## Abstract

Acute respiratory distress syndrome (ARDS) is a critical, life-threatening condition marked by severe inflammation and impaired lung function. Mesenchymal stromal/stem cells (MSCs) present a promising therapeutic avenue due to their immunomodulatory, anti-inflammatory, and regenerative capabilities. This review comprehensively evaluates MSC-based strategies for ARDS treatment, including direct administration, tissue engineering, extracellular vesicles (EVs), nanoparticles, natural products, artificial intelligence (AI), gene modification, and MSC preconditioning. Direct MSC administration has demonstrated therapeutic potential but necessitates optimization to overcome challenges related to effective cell delivery, homing, and integration into damaged lung tissue. Tissue engineering methods, such as 3D-printed scaffolds and MSC sheets, enhance MSC survival and functionality within lung tissue. EVs and MSC-derived nanoparticles offer scalable and safer alternatives to cell-based therapies. Likewise, natural products and bioactive compounds derived from plants can augment MSC function and resilience, offering complementary strategies to enhance therapeutic outcomes. In addition, AI technologies could aid in optimizing MSC delivery and dosing, and gene editing tools like CRISPR/Cas9 allow precise modification of MSCs to enhance their therapeutic properties and target specific ARDS mechanisms. Preconditioning MSCs with hypoxia, growth factors, or pharmacological agents further enhances their therapeutic potential. While MSC therapies hold significant promise for ARDS, extensive research and clinical trials are essential to determine optimal protocols and ensure long-term safety and effectiveness.

## Introduction

Acute respiratory distress syndrome (ARDS) is a rapid and life-threatening condition often caused by severe infection or inflammation that can be characterized by a sudden onset of inflammatory and edematous lung injury, resulting in dyspnea, hypoxemia, decreased lung compliance, and diffuse involvement of the alveolar-capillary unit (1). ARDS can cause fluid accumulation in the lungs, making it difficult for the body to deliver enough oxygen to vital organs. Harsh rapid breathing and wheezing may follow, as well as a severe cough and even a bluish coloration of the skin (2). The injury to the alveoli can be directly caused by conditions such as pneumonia, sepsis, smoke inhalation, and trauma, or indirectly from lung damage associated with multi-organ failure.

A recent study highlighted the long history of ARDS's significant burden on healthcare and its relationship to the fatality rate of up to 50 percent, particularly in the intensive care unit (ICU) (3). The recent coronavirus disease (COVID-19) outbreak has increased the mortality rate among ARDS patients globally. Indeed, ARDS is a deadly form of respiratory disease that frequently affects COVID-19 patients. A past study revealed that one-third of admitted patients have ARDS. Furthermore, three-quarters of COVID-19 patients in the ICU also developed ARDS (4). However, there is no known cure for ARDS due to the heterogeneity and complexity of the underlying causes. Currently, ARDS treatment strategies mainly rely on supportive treatment. Therefore, there is an immediate need for novel therapeutic strategies (3).

Stem cells, especially multipotent mesenchymal stromal/stem cells (MSCs), have immunoregulatory potential to repress inflammation reactions (4). MSCs are derived from various sources, including bone marrow (BM), umbilical cord (UC), adipose tissue (AT), and perinatal tissue (PT), among others. MSCs have shown substantial therapeutic capability in treating different lung diseases. Bone marrow-derived mesenchymal stem cells (BM-MSCs) have been found to be promising in treating acute lung injury (ALI) triggered by lipopolysaccharide (LPS), hyperoxia, pneumonia, and systemic sepsis in animal models (3). Hence, this review extensively discusses various approaches using MSC-based treatment such as direct administration, gene modification, preconditioning, tissue engineering, extracellular vesicles, nanoparticles, natural products, and also the incorporation of artificial intelligence.

## Material and Methods

This is a narrative review; we included the articles with the best matches in Google Scholar, PubMed, Science Direct, and Scopus databases within the last 30 years for each keyword: “multipotent mesenchymal stromal/stem cells”, “acute respiratory distress syndrome”, “treatment strategy”, “direct administration”, “tissue engineering”, “extracellular vesicles”, nanoparticles”, “natural products”, artificial intelligence”, “gene modification”, and “preconditioning”. The articles in the references of the related literature were also included.

## Acute respiratory distress syndrome (ARDS)

### Background

ARDS is a typical response that is triggered by several factors. Beginning with the destruction of the alveolar capillary, it advances through distinct stages including a proliferative stage identified by elevated lung function and cell repair and the fibrotic stage, which indicates the last stage of the acute illness process (5). ARDS can be attributed to infection, chemicals, and trauma due to factors like smoking, alcohol consumption, and bacterial infection on the alveolar epithelium and lung endothelium, which can disrupt the boundary of epithelial or endothelial cells as well as trigger the activation and accumulation of inflammatory cells in the interstitial and bronchoalveolar environment. The enormous inflammatory response leads to alveolar hemorrhage and pulmonary edema, which ultimately leads to failure of the respiratory system (3).

The incidence of ARDS is estimated at around 16 to 78 per 100,000 population and varies between countries, but it tends to increase in patients with underlying medical conditions typically treated in the ICU such as trauma and infection, or with other risk factors including age, race, and gender. Meanwhile, the hospital mortality rate is highly dependent on underlying comorbidities and the severity of the disease. It is estimated that the overall ARDS mortality rate is between 27 and 45% (6). Unfortunately, information is still scarce in Malaysia and there is lack of comprehensive and standardized data collection systems. In addition, ARDS is complex to diagnose and classify. This poses challenges in accurately understanding the incidence and mortality rate of ARDS in Malaysia, impeding the identification of high-risk populations, the development of effective prevention and treatment strategies, and the allocation of healthcare resources. Recent studies suggest that early diagnosis, as well as appropriate supportive care and management of ARDS such as the use of low-tidal volume mechanical ventilation, oxygen therapy, and lung protective strategies, can improve patient outcomes and mortality rates (5,7).

### ARDS and COVID-19

COVID-19, which is caused by the SARS-CoV-2 virus, is a novel condition that emerged in 2020 and has been associated with ARDS in severe cases (8). The SARS-CoV-2 virus primarily affects the respiratory system, although other organ systems are also involved. Lower respiratory tract infection-related symptoms including fever, dry cough, and dyspnea were reported in the initial case series from Wuhan, China. In addition, headache, dizziness, generalized weakness, vomiting, and diarrhea were reported (9). Respiratory symptoms of COVID-19 are extremely heterogeneous, ranging from minimal symptoms to significant hypoxia with ARDS. COVID-19 progression can be divided into three distinct phases, including the early infection phase, in which the virus infiltrates host cells in the lung parenchyma, the pulmonary phase, in which viral propagation causes lung tissue injury as the host immune response is activated, and the inflammatory cascade, which is triggered by pathogen-associated molecular patterns, and damage-associated molecular patterns are exposed during active viral replication and release (10).

According to Tanzadehpanah et al. (11), SARS-CoV-2 can invade epithelial cells, hence many patients infected with SARS-CoV-2 suffer from vascular diseases (VDs) in addition to pulmonary manifestations. Endothelial cells express angiotensin-converting enzyme 2 (ACE2). ACE2 is a biological enzyme that converts angiotensin (Ang)-2 to Ang-(1-7). SARS-CoV-2 uses ACE2 as a cell receptor for viral entry. Thus, this virus promotes the downregulation of ACE2, Ang-(1-7), and anti-inflammatory cytokines, as well as an increase in Ang-2, resulting in pro-inflammatory cytokines. SARS-CoV-2 infection can cause hypertension and endothelial damage, which can lead to intravascular thrombosis.

The inflammatory response plays a crucial role in the clinical manifestations and subsequent complications of COVID-19 (10). The exact pathogenesis of ARDS in COVID-19 remains unclear, but several mechanisms have been proposed (4). It is believed that severe ARDS in COVID-19 patients results from several overlapping processes such as direct viral invasion, lung injury from inflammatory cytokines, and recruitment of activated leukocytes, as well as systemic coagulopathy and multiorgan failure (12). This is supported by case studies demonstrating that histopathological examination reveals diffuse alveolar damage, intra-alveolar leukocyte infiltration, and the presence of virus in the lung (13). Moreover, studies have confirmed the association between ARDS, cytokine storm, and activation of local and systemic inflammation in COVID-19 (14,15).

The mortality of COVID-19 ARDS ranges between 26 and 61.5% in patients admitted into a critical care setting, and in patients who receive mechanical ventilation, the mortality can range between 65.7 to 94% (16). Severe COVID-19 is characterized by viral pneumonia from SARS-CoV-2 infection leading to ARDS. COVID-19 ARDS is diagnosed when someone with confirmed COVID-19 infection meets the Berlin 2012 ARDS diagnostic criteria. ARDS is underdiagnosed in intensive care settings. ARDS develops in 42% of patients presenting with COVID-19 pneumonia, and 61-81% of those requiring intensive care. COVID-19 ARDS causes the typical ARDS pathological changes of diffuse alveolar damage in the lung. Pulmonary thrombosis is common in sepsis-induced ARDS. Coagulation dysfunction also appears to be common in COVID-19. Vascular enlargement is rarely reported in typical ARDS, but has been seen in most cases of COVID-19 ARDS. COVID-19 ARDS appears to have worse outcomes than ARDS from other causes (16).

### Current clinical treatment and challenges

Treatment for ARDS usually involves supplemental oxygen, medication to reduce inflammation or infection, respiratory support such as non-invasive ventilation or invasive mechanical ventilation, and supportive care such as nutrition and bed rest (17). ARDS is potentially fatal if not treated immediately. The exact cause of ARDS is not yet known but the current consensus is that the initial damage to the alveoli will cause inflammation, leading to a greater level of fluid accumulation in the lungs. This can be attributed to several events such as the release of cytokines and other inflammatory mediators and dysregulation of coagulation and capillary permeability. ARDS is a complex condition, and its development is thought to involve a range of mechanisms, sometimes primarily, but not exclusively, immunological reactions (18).

Previous studies reveal various clinical treatment approaches for ARDS. For instance, ventilatory support including invasive and non-invasive mechanical ventilation, tracheotomy, tidal volume size, plateau pressure, positive end-expiratory pressure (PEEP), and rescue therapies as well as pharmacological interventions using glucocorticosteroids, salbutamol, and surfactants, among others (5,7). Mechanical ventilation is used to treat certain ARDS patients only. It has been recommended to ventilate ARDS patients with low tidal volumes when they are intubated. A plateau pressure of less than 30 cm H_2_O is suggested, but a plateau pressure of less than 15 cm H_2_O is advised to be safe. High levels of PEEP are prescribed to patients with mild and serious ARDS conditions. Examples of rescue therapies are the prone position and neuromuscular blocking agents. Tracheotomy is considered in the case of extended mechanical ventilation (7).

Regarding pharmacological interventions, therapy with steroids, especially glucocorticosteroids, was among the only strategies studied thus far in clinical trials that consistently minimized morbidity and mortality rates among ARDS patients (7). However, there are still challenges in the ARDS clinical treatment (7), particularly inefficacious therapies, innate biological diversity, and poor selection of patients for the treatment strategies. A previous study also emphasized that numerous clinical trials on mechanical ventilation and pharmacological treatment for ARDS patients have been performed. Nevertheless, none of them has been fully beneficial to ARDS treatment (7). Therefore, medical practitioners are in dire need of an alternative clinical treatment using a stem cell approach for ARDS patients.

The limited information on the incidence and mortality rate of ARDS in Malaysia impedes the identification of high-risk populations, the development of effective prevention and treatment strategies, and the allocation of healthcare resources. Therefore, efforts should be made to establish centralized data collection systems and enhance the consistency and accuracy of ARDS diagnosis and classification in Malaysia.

## Multipotent MSCs

Research related to MSCs has rapidly progressed in recent years. Therefore, there is a significant need for the standardization of terminology and important characterization. Viswanathan et al. (19) stated that the International Society of Cell and Gene Therapy (ISCT) and the International Standards Organization's (ISO) Technical Committee (TC) on Biotechnology have published the ISO standardization focusing on biobanking of MSCs from two different sources of tissues, including UC-MSCs and BM-MSCs. The functional meaning of mesenchymal stromal cells *versus* mesenchymal stem cells is described in ISO/TS 22859 for umbilical cord tissue mesenchymal stromal cells and in ISO 24651 for bone marrow-derived mesenchymal stromal cells. For the former, a strong matrix of *in vitro* assays is needed, whereas the self-renewal and differentiation capabilities of MSCs *in vivo* and *in vitro* confirm the latter. Bianco (20) highlighted that mesenchymal stem cells are not always stem cells and mesenchymal. Alternatively, they can also be multipotent stromal cells, mesenchymal stromal cells, and medicinal signaling cells, which convey different meanings. However, all different terms have one constant acronym, which is MSC.

Based on Ghabriel et al. (21), MSCs are defined as multipotent cells that have the ability to self-renew and differentiate into a wide variety of cell types including muscle cells, cartilage cells, tendon cells, bone cells, fibroblasts, and adipocytes. MSCs have been found to have broad bioactivities, including repair, immunomodulation, increased alveolar fluid clearance, and regulation of pulmonary vascular endothelial permeability (22). They are important in tissue regeneration and wound healing and can be used to treat many disorders, including autoimmune diseases, diabetes, and leukemia. Existing strategies for ARDS treatment have been limited in their ability to improve clinical outcomes. Increasing evidence suggests that MSCs may serve as a potent immunomodulatory intervention for ARDS therapy due to their ability to reduce proinflammatory cytokines and up-regulate anti-inflammatory cytokines (23). Additionally, MSCs can interact with other immune cells to limit organ damage, inhibit inflammation, and stimulate tissue regeneration (24). Through their low immunogenicity and tri-lineage differentiation capabilities, MSCs present unique advantages as therapeutic agents. Preclinical trials indicate that MSCs secrete several types of cytokines, modulate macrophages, and influence the balance between pro- and anti-inflammatory cells (25).

### Isolation and characterization

Isolation and characterization of MSCs allow us to study the properties and behavior of these cells to better understand their roles in regenerative medicine. This includes testing cell growth potential, determining the cell types into which it is likely to differentiate, determining its immunomodulatory potential, and assessing its surface markers. BM-MSCs are one of the major sources of stem cells that were initially identified in bone marrow (26). A past study reveals that BM-MSCs were predominantly used in many novel drugs up until 2008 (27). However, there are several other available sources for MSC isolation not limited to muscle, umbilical cord, perinatal tissue, and adipose tissue (27). Past studies also demonstrated that MSCs derived from distinct tissues have numerous similar characteristics. Nevertheless, characteristics of MSCs including proliferative capacity, secretion of growth factor, differentiating ability, and hemocompatibility greatly differ according to culture conditions and tissue source (27).

Peripheral blood-derived MSCs are a type of adult stem cell that can be isolated from the peripheral blood of human donors. Peripheral blood contains a heterogeneous population of stem cells capable of giving rise to MSCs with multipotent properties. Although it contains a small number of circulating MSCs, it can also proliferate and differentiate *in vitro*. Secondly, umbilical cord blood contains hematopoietic cells and a number of other non-hematopoietic populations that give rise to MSCs. Finally, adipose tissue is also a potential source of MSCs. Characterization of MSCs enables researchers to understand a cell's potential and develop improved therapies. MSCs are commonly characterized by the presence of several biological markers including CD73, CD90, and CD105 (28). Such markers allow the identification and isolation of populations of MSCs for further study.

The different levels of incompatibility of MSCs with human blood are known to jeopardize safety and efficacy. MSCs can initiate the coagulation cascade in response to various conditions, including passage number, tissue origins, cell dosage, and expression of pro-coagulant factors including tissue factor (TF) and phosphatidylserine (PS) on MSCs (29). TF, for instance, is a significant factor affecting the hemocompatibility of cell products. Under certain circumstances, MSCs exhibit TF, which could potentially promote a prothrombotic environment (29). Thus, this factor must be regularly checked in all medications that are administered intravenously. In addition, the infusion of pro-coagulant TF-expressing cell products such as MSCs, hepatocytes, and pancreatic islets can result in innate immune-attack that could deteriorate the safety, efficacy, and engraftment of cells (27). A meta-analysis has demonstrated that bone marrow MSC infusion is safe. Different MSC products display varying levels of highly pro-coagulant TF and may adversely trigger the instant blood-mediated inflammatory reaction (IBMIR). Suitable strategies for assessing and controlling hemocompatibility and optimized cell delivery are crucial for the development of safer and more effective MSC therapies (27).

In addition, it has also been found that the process of freeze-thawing of MSCs can potentially impact the efficacy of MSCs, especially viability and functionality. Freeze-thawing is a process used to preserve cells for long-term storage and transportation. However, this process may cause cellular damage, loss of potency, and altered immunomodulatory properties of MSCs. Several factors, such as freezing medium, cooling rate, storage temperature, thawing method, and post-thaw culture, may influence the outcome of freeze-thawing of MSCs. Several studies have investigated the impact of cryopreservation and freeze-thawing on the therapeutic properties of MSCs (30), suggesting that cryopreserved MSCs may display impaired immunomodulatory and therapeutic properties compared to fresh MSCs (31). Therefore, it is important to optimize and standardize the freeze-thawing protocols for MSCs to ensure their quality, safety, and efficacy for clinical applications.

In summary, understanding the functional properties of MSCs is pivotal for enhancing the safety and efficacy of their therapeutic applications, and addressing these factors ensures the development of safer and more potent MSC therapies, ultimately improving patient outcomes.

### Therapeutic potential of MSCs

**Figure 1:**
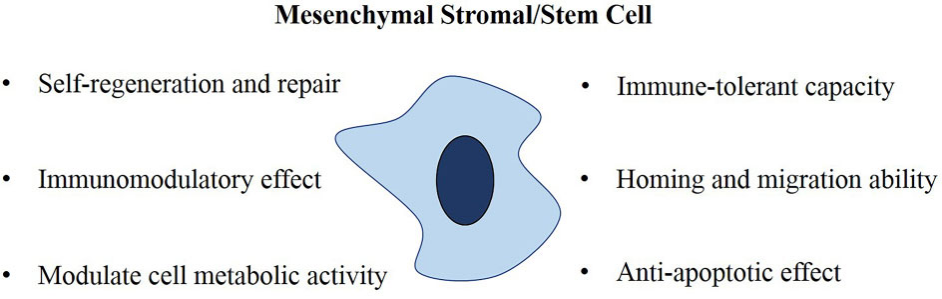
Characteristics of mesenchymal stromal/stem cells (MSCs).

MSC therapy is being explored as a promising new strategy for the treatment of ARDS (32). MSCs possess several unique properties including self-regeneration and repair, immunomodulatory effect, anti-apoptotic effect, immune-tolerant capacity, homing, and migration ability, and modulate cell metabolic activity as illustrated in Figure 1. These properties made MSCs a popular choice for stem cell research and clinical applications in recent years.

Generally, MSCs have the capacity for self-renewal and the ability to differentiate into almost any tissue type. They can be isolated from various adult tissues, including bone marrow, adipose tissue, and umbilical cord tissue, and are generally easier to be accessed compared to other types of stem cells (33). Additionally, MSCs have been found to possess anti-inflammatory and immunomodulatory properties that protect host tissue upon transplantation, with few side-effects associated with their use (34). These advantages make MSCs an attractive choice for regenerative medicine applications.

A wealth of research supports the idea that intravenous, intranasal, and intratracheal route of MSC administration could lead to significant improvements in inflammatory gene expression and lung function, reducing mortality in patients with ARDS (32). Furthermore, a number of cytokines secreted by MSCs may modulate immune responses and aid in the recovery of damaged tissues. For example, MSCs secrete anti-inflammatory mediators such as tumor necrosis factor-alpha, interleukin-10, and transforming growth factor-beta 1, which serve to dampen inflammation and reduce apoptosis of epithelial cells in ARDS (35).

In addition, ARDS-mediated inflammation can be suppressed when MSCs are transplanted to the damaged lungs via an intratracheal route. Specifically, MSCs can reduce the severity of ARDS-associated pulmonary edema and sepsis, as well as promote neutrophil apoptosis, reduce inflammatory cell infiltration, and improve vascular integrity (36). The immunomodulatory effect of MSCs has been further supported by studies showing that MSC-based therapies reduce mortality rates of ARDS and improve organ damage after being challenged by LPS. There is increasing evidence suggesting that MSCs have the potential to serve as an effective therapeutic modality for ARDS due to their capability to modulate inflammation, improve lung function, and reduce mortality. Further research and clinical trials exploring multi-functional properties of MSCs are warranted to improve understanding of potential applications of MSCs in ARDS treatment.

The potential of MSC therapy for ARDS presents a promising avenue for tackling the challenges encountered by ARDS patients, especially those severely affected by COVID-19. Existing treatment options are limited, and mortality rates remain high. This is compounded by issues such as a lack of standardized modes of delivery and concerns regarding cell product quality, including potential effects on patient coagulopathy and the need for thromboprophylaxis (37). However, to fully harness these possibilities, a more in-depth exploration is required through additional preclinical and clinical studies. These investigations are crucial to refine and validate the efficacy and safety of MSC-based strategies for ARDS. This review comprehensively explores diverse approaches to MSC-based treatment, ranging from direct administration to gene modification, preconditioning, tissue engineering, extracellular vesicles, nanoparticles, natural products, and even the integration of artificial intelligence.

## MSC treatment strategies for ARDS

The use of MSC therapy has been explored as a potential treatment strategy for a wide range of diseases, including ARDS. This section evaluates recent studies and reviews of various clinical and pre-clinical applications of MSCs for ARDS. It also discusses efficacy findings as well as the status of clinical studies and patient outcomes.

### Direct administration

Over the last decade, an increasing number of pre-clinical studies have demonstrated that the direct administration of MSCs has therapeutic potential for ARDS, including anti-inflammatory, anti-apoptotic, and regenerative effects. MSCs successfully mediated anti-inflammatory effects in a rat model of ARDS, significantly reducing levels of interleukin-6 (IL-6) and improving pulmonary histological changes (38). Devaney et al. (39) also noted that mouse BM-MSCs administered intravenously and intratracheally to mouse models of sepsis-induced lung injury caused dramatic changes in lung histopathology, significantly reduced inflammation by regulating inflammatory cytokines, reduced bacterial load in bronchoalveolar lavage fluid (BALF), suppressed neutrophil infiltration, and improved the survival rate. Likewise, BM-MSCs administered intravenously managed to delay the development and reduce the severity of ARDS and suppressed the systemic concentration of TNF-α, a pro-inflammatory cytokine (40).

To date, several clinical trials have also been completed to assess the safety and efficacy of MSC therapy for ARDS in humans. The results from these studies indicate that MSCs appear to be safe, as no major adverse events have been associated with the administration of MSCs for ARDS.

In a phase I clinical trial, nine patients with ARDS were intravenously administered with a single dose of allogeneic MSCs up to 10 million cells/kg body weight, in addition to conventional medical treatment. MSCs were well tolerated in patients with moderate-to-severe ARDS for up to six months of follow-up after administration and led to a significant reduction of plasma surfactant protein D (plasma SP-D) and a non-significant reduction of IL-6 (41). Likewise, a small RCT by Zheng et al. (42) reported a supporting observation where there was a significant reduction in the concentration of plasma SP-D and pro-inflammatory cytokines including IL-6 and IL-8.

Additionally, in a phase 2 START trial with 60 ARDS patients, Wick et al. (43) reported that MSCs induced a reduction of biological markers of inflammation like BAL Ang-2, IL-6, and TNF receptor-1 concentrations suggesting a potential benefit in ARDS patients. Hsueh et al. (44) also highlighted several studies that tested the intravenous infusion (IVF) approach using UC-MSCs. They revealed that the UC-MSCs significantly minimized the release of numerous inflammatory cytokines *in vivo* as well as alleviated hypoxemia among patients exhibiting serious COVID-19-related ARDS conditions. Intriguingly, a single dose of IVF-UC-MSCs can improve the initial severity of ARDS.

Likewise, Wang et al. (45) claimed that IVF is the best approach of MSC administration for ARDS patients. The direct interaction between MSCs and blood halts the cytokine storm. The study also emphasized that the infusion of MSCs using the intravenous route demonstrated substantial benefits toward pulmonary function. However, there is a risk of the occurrence of dose-dependent pulmonary emboli or infarctions due to the intravenous administration of MSCs (25). In a case report, Jung et al. (46) also highlighted that the administration of human adipose tissue-derived stem cells (hASC) via intravenous route led to pulmonary embolism.

In summary, the available evidence suggests that direct administration of MSCs, especially by intravenous routes, is possibly a safe and effective treatment for ARDS. Although preclinical and clinical studies have demonstrated the MSCs potential, more studies are needed to further assess the efficacy and safety of MSC therapy for human patients.

### Gene modification

Presently, researchers were unable to make a conclusive deduction on the therapeutic potential of MSCs in ARDS patients due to inadequate number of randomized phase III/IV clinical trials. Thus, the genetically modified MSCs that increase the production of beneficial trophic cytokines or other genes to enhance the therapeutic potentials of MSC products are widely studied using different preclinical models.

In the genetic engineering of MSCs, approaches such as viral and non-viral transfection are generally implemented. Non-viral transfection can be categorized into physical methods, including electroporation and ultrasound sonoporation, and chemical methods such as liposomes. Non-viral transfection has several advantages, including higher scalability, low cost of manufacturing, and low risk of serious immune response, but also has some limitations. In contrast, viral transduction is a highly efficient approach used to incorporate exogenous genes into MSCs and does not affect the self-regenerating and differentiation ability of the progeny (47). The commonly used viral vectors in this approach are the lentiviral vector, adenoviral vector, and adeno-associated virus (AAV). However, this approach has some limitations, including the risk of triggering oncogenes, which might develop into tumors, and the ability of viral vectors to induce severe immune responses while debilitating the stability of the transgene (48). Thus, previous studies have transferred therapeutic transgenes into human-induced pluripotent stem cell (iPSC)-derived MSCs prior to the derivation of MSCs. This approach reduces the mutation triggered by insertion and it allows steady expression of transgenes during the extended development process (49).

Moreover, earlier studies discuss that genetic modification of MSCs enhances homing properties, anti-inflammation, and anti-apoptosis, restores the barrier between alveoli and capillaries, and allows the cells to survive in unfavorable conditions (47). Hence, several genes such as heme oxygenase-1 (*HO-1*), chemokine receptor 4 (*CXCR4*), and *ACE2*, among others were thoroughly investigated using preclinical ARDS models (47). HO-1 is a type of protein associated with stress reactions with anti-apoptotic, anti-inflammatory, and antioxidative characteristics. This gene also shields cells from injury and maintains homeostasis balance in different chronic stages. Chen et al. (50) found that altering *HO-1* in MSCs alleviates inflammation and oxidative stress triggered by LPS in pulmonary microvascular endothelial cells (PVECs). The transwell system was used to coculture PVECs damaged with LPS, and MSCs were injected with *HO-1* gene. The researchers performed additional experiments on the protective effects of BM-MSCs showing excessive stimulation of *HO-1* using lentiviral vectors in the LPS-triggered ALI rat. HO-1-MSCs improved survival rate, attenuated lung pathological impairments, and suppressed the inflammatory reaction (51).

Apart from that, the Clustered Regularly Interspaced Short Palindromic Repeats (CRISPR)/Cas9 is an approach that is commonly used in genetic modification. CRISPR/Cas9 approach is considered more efficient than other gene modification techniques, including effective quasi-active transcription nuclei (TALENS) and zinc finger nucleases (ZFNs), due to its cost-efficiency as it can manage numerous gene targets concurrently. It also has the ability to modify the genome with high sensitivity and specificity (52). The CRISPR/Cas9 approach develops a target-specific double-stranded break in a genome using a complex of Cas9 nuclease and a short RNA. The gene mutation can be reversed by the addition of a new sequence that is controlled by the homology-directed repair. Furthermore, this approach can be implemented to fix mutations in genes, introduce a knock-in or knock-out mutation, or knock down the expression of a particular gene. Hence, this approach is used to improve the therapeutic efficacy of MSCs as well as alter their cytokine level and production of various growth factors (53).

In summary, genetic modification on MSC-based strategies has proven to provide favorable outcomes in preclinical animal models with ARDS. While there has been some discussion around the use of genetically modified MSCs for translational purposes, it remains a topic of uncertainty and scrutiny. Researchers are currently working to establish regulatory compliances for these methods and protocols, which has proven to be a challenge. It is important that future studies address the potential drawbacks associated with genetic modification for MSC-based strategies, in order to further develop this area of research.

### Preconditioning

The adaptive technique of preconditioning MSCs aims to prepare them for survival in difficult conditions and improve their regulatory capacity towards local immune responses. To improve MSC-based treatments for ARDS, a number of preconditioning strategies have been developed, including preconditioning with hypoxia, serum from ARDS patients, N-acetylcysteine, transforming growth factor beta (TGF-β), and three-dimensional (3D) culture.

Preconditioning with hypoxia prepares MSCs for the hypoxic conditions found in ischemia microenvironments, lowering hypoxia-induced cellular death. The results of the ARDS preclinical rat model of ischemia/reperfusion-induced lung injury show that hypoxic MSCs can quickly migrate into extravascular lung tissue and adhere to other inflammatory or structure cells, thereby attenuating ischemia/reperfusion (I/R) lung injury via antioxidant, anti-inflammatory, and anti-apoptotic mechanisms (54). Treatment with hypoxia-conditioned MSCs also improves pulmonary respiratory functions and reduces inflammation with production of pro-fibrotic factors. Pre-activation with serum obtained from ARDS patients improves MSC anti-inflammatory activity, proven by an increase in IL-10 and IL-1-RN secretion. Furthermore, after transplantation into an ARDS animal model, serum-pre-activated MSCs are more successful in lowering lung injury scores, inflammatory cell counts, and pulmonary edema (55).

A study has demonstrated that hypoxia augments the therapeutic characteristics of both porcine and human MSCs. Yet, short-term 2% hypoxia offers the greatest benefit overall, exemplified by the increase in proliferation, self-renewing capacity, modulation of key genes, and improvement of the inflammatory milieu compared to normoxia. These data are important for generating robust MSCs with augmented functions for clinical application (56).

N-acetylcysteine (NAC) is a glutathione precursor that protects against the harmful effects of reactive oxygen species (ROS) by scavenging free radicals and providing substrates for antioxidant enzymes. Preconditioning with NAC *in vitro* has been shown to eliminate cellular ROS, boost cellular glutathione levels, promote cell adhesion and spreading, and improve the cell's antioxidant capacity to defend against redox imbalances. When treated human embryonic MSCs (hMSCs) were transplanted into nude mice with bleomycin-induced lung injury, they significantly influenced the repair process and fibrosis. NAC treatment of hMSCs could be an optimized therapeutic method to enhance cell transplantation and lung injury treatment (57).

The preconditioning of MSCs with low-level transforming growth factor-1 (TGF-1) resulted in enhanced expression of fibronectin, a key component of the extracellular matrix. The results of a rat model of LPS-induced ALI revealed that an increased number of MSCs were found in the lung two weeks after transplantation, indicating that TGF-1-treated MSCs may improve their long-term therapeutic impact on tissue repair (58). A study also shows that activation of allogeneic MSCs through incubation in an environment previously exposed to MSCs may induce stronger immunomodulatory effects in patients compared with an infusion of non-activated MSCs (59).

MSC culture in a 3D microenvironment is a unique preconditioning method that mimics the physiological or pathological milieu in which the cells would reside following transplantation. According to Bartosh et al. (60), 3D culture of MSCs in spheroids was more effective than an adherent culture of MSCs in attenuating neutrophil activity and reducing proinflammatory cytokines in a mouse model of peritonitis, indicating that 3D MSC culture is a promising approach for diseases with unresolved inflammation. Additional research has discovered that adipose-derived stem cells subjected to short-term spheroid formation prior to monolayer culture had superior regeneration potential by boosting their chemotaxis, angiogenesis, and stemness features, enhancing their repair capacity for clinical use (61).

Research by Kudinov et al. (62) also discovered that three inhalation infusions of the freeze-dried secretome from 2D- and 3D-cultured placental multipotent mesenchymal stromal cells (MMSC) and lung fibroblasts (LFB) protected mice from death, repaired the histological structure of injured lungs, and reduced fibrin deposition. On certain measures, the 3D-MMSC secretome showed a more significant effect on lung recovery than the 2D-MMSC and LFB-derived secretome.

### Tissue engineering

Tissue engineering has shown great potential for tissue repair in the treatment of numerous diseases, including orthopedic diseases, degenerative diseases of the skeletal system, and musculoskeletal injuries and associated conditions (63). Tissue engineering using MSCs has emerged as a promising strategy for the treatment of ARDS. MSCs can differentiate into various cell types and possess immunomodulatory properties, making them an attractive candidate for tissue engineering applications.

One of the popular tissue engineering approaches is the incorporation of biomaterials with MSCs, which has been proven to significantly enhance cellular viability following extra-vascular transplantation, thereby playing a crucial role in the suppression and prevention of graft-*versus*-host disease. For instance, the assembly of MSCs and hydrogels into spheroids has shown to promote the secretion of endogenous trophic factors and extracellular matrix, elevate cytokine and immunomodulatory paracrine factor levels, and mitigate the inflammatory response induced by LPS (64). MSC spheroid-loaded collagen hydrogels were able to simultaneously promote neuronal differentiation and suppress the inflammatory reaction through the phosphatidylinositol 3-kinase (PI3K)/Akt signaling pathway by regulating the number of aggregated cells (65). Additionally, in recent years, researchers have explored other tissue-engineering strategies using MSCs for the treatment of ARDS, including 3D-printed scaffold systems, MSC sheets, and mechanical stimulation of MSCs.

The 3D-printed scaffold is a biomimetic structure that mimics the architecture of the lung tissue and provides a support structure for the growth of cells. Researchers have demonstrated the potential of 3D-printed scaffold systems combined with MSCs for the treatment of ARDS in preclinical studies. In an *in vitro* study, lung-resident MSCs (L-MSCs) were 3D-cultured into the bioprinted lung ECM hydrogel. This is a good strategy to better understand different factors affecting their therapeutic potential. Cells were found to have significantly increased adhesion capacity, inducing contraction within the 3D hydrogel indicating that the cells actively interacted with the scaffold and were able to modulate inflammatory mediators (66). Another tissue-engineering strategy for the treatment of ARDS involves the use of MSC sheets. MSC sheets are a layer of cells that can be easily transplanted onto the lung tissue. Researchers have demonstrated the potential of MSC sheets for the treatment of ARDS in preclinical studies. For instance, a study performed by Iwata et al. (67) examined the safety of autologous PDL-derived cell sheets and validated its regenerative potential. The researchers found improvements in periodontal health indicators such as reduced probing depth, increased clinical attachment, and enhanced radiographic bone height observed in all 10 cases over a 55-month follow-up period without any severe adverse events.

Mechanical stimulation of MSCs is another tissue-engineering strategy for the treatment of ARDS. Mechanical stimulation can enhance the differentiation potential and immunomodulatory properties of MSCs, making them more effective in treating ARDS. Researchers have explored various mechanical stimulation techniques, including stretch, compression, and shear stress, for the treatment of ARDS. In an *in vitro* study of pulmonary microvascular endothelium barrier injuries induced by LPS, the mechanical stretch of MSCs affected the morphology, proliferation, and production of inflammatory mediators, reduced paracellular permeability of lung endothelium induced by LPS, attenuated apoptosis, and restored intercellular junction proteins to maintain the integrity of the pulmonary microvascular endothelium barrier. Another study found that prolonged compression loading of hMSCs stimulates the development of mature integrin α5-dependent 3D-matrix adhesions (3DMAs), which enhances the osteogenesis of hMSCs (68).

In conclusion, MSC-based tissue-engineering strategies have shown great promise for the treatment of ARDS. Preclinical studies have demonstrated the potential of 3D printed scaffold systems, MSC sheets, and mechanical stimulation of MSCs for the treatment of ARDS, and clinical trials have shown the safety and efficacy of MSC therapy. With continuous research and clinical trials, it may be possible to unlock even more potential benefits and new applications from this promising therapeutic option.

### Extracellular vesicles

Extracellular vesicles (EVs) are small membrane-bound or nuclear microparticles released by eukaryotic cells. As a highly heterogeneous population, EVs can induce a complex response. They are generally divided into two categories: ectosomes and exosomes. Ectosomes are formed through the plasma membrane's direct outward budding in the 50- to 1000-nm size range. Exosomes originate from endosomes with size of 100 nm on average. EVs can transport a wide range of cargoes, including lipids, RNAs, several protein species, and even organelles such as mitochondria. These substances have the ability to modify the epigenetic environment and protein expression within target cells (69).

EVs have shown therapeutic potential and play critical roles in intercellular communication between MSCs and damaged cells, which may endow them with the ability to transfer bioactive contents between donor and target cells. EVs communicate with target cells via receptor-mediated binding. In the face of limited treatment options for ARDS, mesenchymal stem cells-derived EVs (MSC-EVs) have lately shown promise. MSC-EVs exhibit anti-inflammatory effects, cell injury repair, alveolar fluid clearance, and microbial clearance, which are similar to MSC-based therapy. MSC-EVs' potent therapeutic efficacy and biocompatibility have made them a viable treatment option for ARDS (69).

MSC-EVs' anti-inflammatory effects are primarily associated with their ability to modify immune cell levels, which is related to the recruitment of anti-inflammatory neutrophils to lung tissue (69). Furthermore, Morrison et al. (70) showed that MSCs increase the phagocytic macrophage phenotype via EV-mediated mitochondrial translocation, which results in anti-inflammatory benefits. MSC-EVs also repair injured microvascular endothelial and epithelial cells, which are the leading cause of death for ARDS patients. Hu et al. (71) discovered that MSC-EVs protected lung microvascular endothelial cells from inflammatory injury. MSC-EVs exhibit an angiogenic effect and are also able to reduce mitochondrial dysfunction, thereby restoring pulmonary microvascular endothelial cell damage. Mitochondrial transfer alleviates ARDS and stimulates the recovery of the alveolar epithelial-capillary barrier, which is required to restore alveolar epithelial cell activities for lung tissue repair (72). MSC-EVs' anti-apoptotic and antioxidant activity make them effective for alveolar epithelial tissue regeneration.

EVs released by MSCs increase alveolar fluid evacuation, resulting in the healing of the injured lung tissue in severe ARDS. MSC-EVs have the ability to transfer intercellular components including fibroblast growth factor (FGF) and keratinocyte growth factor (KGF), which can upregulate alveolar fluid transport and restore lung protein permeability.

Microbe clearance is also crucial for the treatment of bacteria- or virus-induced ARDS because microbes can cause significant inflammation and tissue death in the lungs (69). MSC-EVs can transport immunoregulatory substances, including proteins, RNAs, and lipids, to recipient cells and have a regulatory function similar to MSCs (73). To produce antibacterial effects, active antibacterial compounds such as LL-37 and -defensin-2 (BD2) can be loaded into EVs. MSC-EV therapy can boost alveolar macrophage phagocytosis, which improves microbiological clearance (74). In the instance of viral infection-induced ARDS, the underlying mechanism of MSC-EVs inhibits viral multiplication and suppresses the cytokine storm (75).

In conclusion, the anti-inflammatory, cell repair, alveolar fluid clearance, and microbial clearance properties can help to alleviate ARDS (74). MSC-EVs have therapeutic effects similar to MSCs but without the risks associated with MSCs. MSC-EVs have pro-regenerative and targeting properties, as well as the ability to transfer various substances to the recipient cells.

### Nanoparticles

Nanoparticles have been extensively investigated as an innovative drug delivery platform for almost every disease including cancers and tissue damage, over the last few decades (76). For example, gold nanorods/nanostars are well recognized for their photoacoustic and photothermal properties (77). They can absorb light at distinct wavelengths, making them potentially valuable for skin cancer hyperthermic treatments and diagnostic imaging applications (76). Nanoscale particles have unique physicochemical properties that can help to improve the physical and biological properties of drugs in terms of solubility, selectivity, efficacy, pharmacokinetics, and toxicity (78). They also help to overcome challenges such as stability, bioavailability, and systemic distribution of long-acting nanocarriers (79).

The usage of functional parts of cells and their combination with nanomaterials facilitate effective therapeutic drug delivery. Nanoparticles coated with cell membranes have a synthetic nanoparticulate core that is camouflaged by a natural cell membrane layer. These novel biomimicking nanoparticles are designed to have a variety of properties, including possible immunogenicity, prolonged circulation time, and selective targeting. Synthetic nanoparticles coated with membranes derived from MSCs have been employed as nanocarriers with similar promising properties (76).

In ARDS, due to severe endothelial cell destruction, drug delivery to deeper pulmonary tissues is required. The low resolution, rapid clearance, short half-life, and ineffective delivery of drugs to the target organs have limited the efficacy of pharmacotherapies. According to Sababathy et al. (80), the application of stem cells as biological carriers may lead to breakthroughs as part of nanoparticle administration strategies for medical treatment, especially for cancers. This is because nanoparticles have unique advantages including prolonged drug release, non-toxicity, amplified drug delivery, high encapsulation, biocompatibility, and high drug loading capacity (79).

Several drug-delivery systems have been developed and tested on ARDS experimental models in recent years to overcome limitations. For example, combining nanostructure delivery systems with drugs and bioactive molecules enables uniform drug distribution and internalization in well-aerated alveoli and targeted drug-delivery, along with few adverse drug reactions (79). Furthermore, rod-shaped nanoparticles linked to the red blood cells (RBC) surface via non-covalent interaction have been found to increase nanoparticle accumulation in the lungs (81).

To summarize, nanoparticles have the potential to be used to treat ARDS. Combining MSCs with nanomaterials has become a promising approach to facilitate the effective delivery of therapeutic agents. Stem cell membrane-coated nanotherapeutic technologies will provide complementary strategies for the treatment of numerous diseases with nanomedicine in the future. With continuous study and clinical trials, this promising therapeutic option may show more potential advantages.

### Natural products

Many pre-clinical and clinical studies have found natural products to be effective in halting several inflammatory pathways related to ALI and ARDS at the cellular level. This is particularly important considering that there are no effective pharmacological agents to minimize ARDS or alleviate its symptoms (1,65). Anti-inflammatory drugs that are used in current clinical settings exhibit various drawbacks, including adverse reactions and high costs. Therefore, natural products that are more easily accessible, stable, and less toxic appear to be a good alternative. As such, phytopharmaceuticals are a constantly growing research interest, especially in the South Asian region of the world where natural products have already been extensively utilized for traditional treatment of various illnesses including inflammation (82). Studies use natural products to find bioactive compounds, which can boost the development of new drugs for the treatment of inflammatory diseases (83). Examples of naturally existing bioactive compounds are alpinetin (84), ginsenoside Rg5 (83), honokiol (85), and protocatechuic acid (PCA) (83), among others.

For instance, alpinetin is a type of natural flavonoid that can be obtained from the seeds of *Alpinia katsumadai Hayata*. It halts the process of adding phosphoryl group to the I*κ*B*α* protein and subsequently limits NF-*κ*B activation. Another study found that alpinetin is able to hinder the phosphorylation of p38 and ERK-related pathways (84). The research by Patel et al. found that ginsenoside Rg5, which is a derivative of ginseng, notably reduces inflammation in ARDS models by controlling the TLR4 receptor (83). Honokiol is a small molecule of the magnolia bark extract (MBE) that shows anti-angiogenic, anti-inflammatory, and anti-tumor properties (85).

To date, the efficiency of modern drugs such as prednisone, prednisolone, and ulinastatin in treating ALI or ARDS is quite inadequate because these drugs have serious side effects, including gastrointestinal inflammation and allergic responses (86). In contrast, natural products have various pharmacological characteristics such as anti-inflammatory, antioxidant, antibacterial, anticancer, and immunomodulatory effects as well as mild negative effects (85). Hence, natural products can be used with MSC-based strategies to enhance the functions of MSCs in treating ARDS patients. For example, it was found that curcumin, a turmeric rhizome extract, has protective effects on the cellular process, as it enhances the survival of bone marrow MSCs by improving the function of mitochondria and enriching the G2/M and S stages of the cell cycle. According to Alagesan et al. (87), incorporating curcumin with hypoxia pre-conditioned MSCs ultimately improved the healing of a cutaneous injury in a mouse model.

To summarize, several studies have been executed to investigate natural products’ abilities in treating ARDS, showing that the efficacy of natural products is more satisfactory than that of modern drugs. Therefore, the use of natural products in MSC-based strategies to treat ARDS patients can result in promising outcomes and inspire the development of novel drugs.

### Artificial intelligence (AI)

With the advent of artificial intelligence (AI) in the healthcare sector, machine learning algorithms are widely trained to identify hidden trends or patterns in various datasets, including electronic health records (88). AI is also implemented to achieve different goals, including the diagnosis of ARDS (89) and prediction of severity and mortality among ARDS patients (90). For instance, Yang et al. (89) implemented various classifier algorithms such as L2-regularized Logistic Regression (L2-LR), Single Hidden Layer Feedforward Neural Network (SLP-FNN), AdaBoost, XGBoost, and a conventional non-invasive classifier to determine the presence of ARDS. The classifier algorithm XGBoost appeared as the best predictive model with an accuracy of 85.89%.

In MSC-based treatments, AI is used to identify complex and important compounds, including sequence of proteins, structure of molecules, and stability between targeted compounds and cell receptors. The data are used to develop prediction models. The automated system for the development of small compounds is useful for various purposes, including medicine, solar power, and polymer chemistry. However, clinical trials will be needed to test drugs discovered using AI in the near future. Likewise, a previous ARDS-based study suggested that AI tools can be incorporated to enhance MSC-based treatment strategies. The author stated that AI can be used to select the ideal MSC, increase the expansion and production of MSCs with optimal conditions in bioreactors, and perform sufficient functional testing prior to clinical practice (32).

Furthermore, because ARDS is an acute and serious disease with sudden onset, medical practitioners face obstacles in cultivating and preparing MSCs on time once the patient has been admitted to the hospital. MSCs are required to be prepared and cryopreserved in advance. Other factors such as the isolation technique, the passage number, and the growth media also affect the biological characteristics of MSCs. Therefore, it is important that MSCs are prepared for treatments as soon as possible and that ways to expedite the production of high-quality MSCs are explored, including isolation and characterization processes. Machine learning algorithms are used to characterize MSCs, and thus enhance the operation and outcome of MSC production (88).

Marklein et al. (74) applied visual stochastic neighbor embedding (viSNE), a type of machine learning approach to characterize MSCs according to their cell structures. The study revealed a correlation between several IFN-γ exposed subpopulations of MSCs and their capability to halt the stimulation of CD4+ and CD8+ T-cells. The model was also found to be efficient in predicting the immunosuppression of other MSC lots. *In vitro* cell expansion is a very crucial step in every cell-based therapy. Hence, a more rapid and cost-saving method is required. In a past study, Enes et al. (88) highlighted the application of Random Forest (RF), a machine learning algorithm for predicting the population doubling time of MSCs, considering the respective donor-related features. This approach helps to automate the process of MSC cell expansion to save time and costs.

In the era of precision medicine, AI plays a significant role in the development of precise individualized medicines for patients to enhance their treatment responses. The heterogeneity of a patient has a major impact on the effectiveness of MSC treatment, particularly in the case of ARDS. Thus, it is vital to predict and identify patients who show higher chances of responding to the optimized treatment, which was followed by MSC manufacturing and pre-conditioning. Earlier clinical research found that MSCs are useful to treat patients with different lung diseases, ranging from chronic to acute conditions. Nevertheless, it is highly questionable whether MSC-based cell therapy is able to remodel damaged tissues found in patients with final-stage chronic lung diseases. This is because MSCs are able to function based on secreted factors and they will usually be eliminated from the lungs after a few days. Hence, there is a higher possibility for ARDS patients to respond well to this optimized treatment (88). Additionally, the advent of AI and tissue engineering has resulted in the development of 3D scaffold biomaterials. These biomimetic scaffolds favor the administration of MSCs in terms of adhesiveness and viability. Moreover, the essential components produced by MSCs are preserved. Hence, this approach lengthens the crucial period in clinical treatment (49).

In short, various approaches implement AI to optimize MSC-based strategies, such as for understanding the significant components of MSCs, enhancing the quality of MSC manufacturing and products, and identifying suitable MSCs for clinical trials. AI is also employed in MSC-based strategies to promote personalized care for ARDS patients and, when integrated with tissue engineering, to prolong the effective period of treatment. Further advancements using AI in MSC-based strategies to treat ARDS patients might lead to promising innovations in the future.

## Conclusions

**Figure 2:**
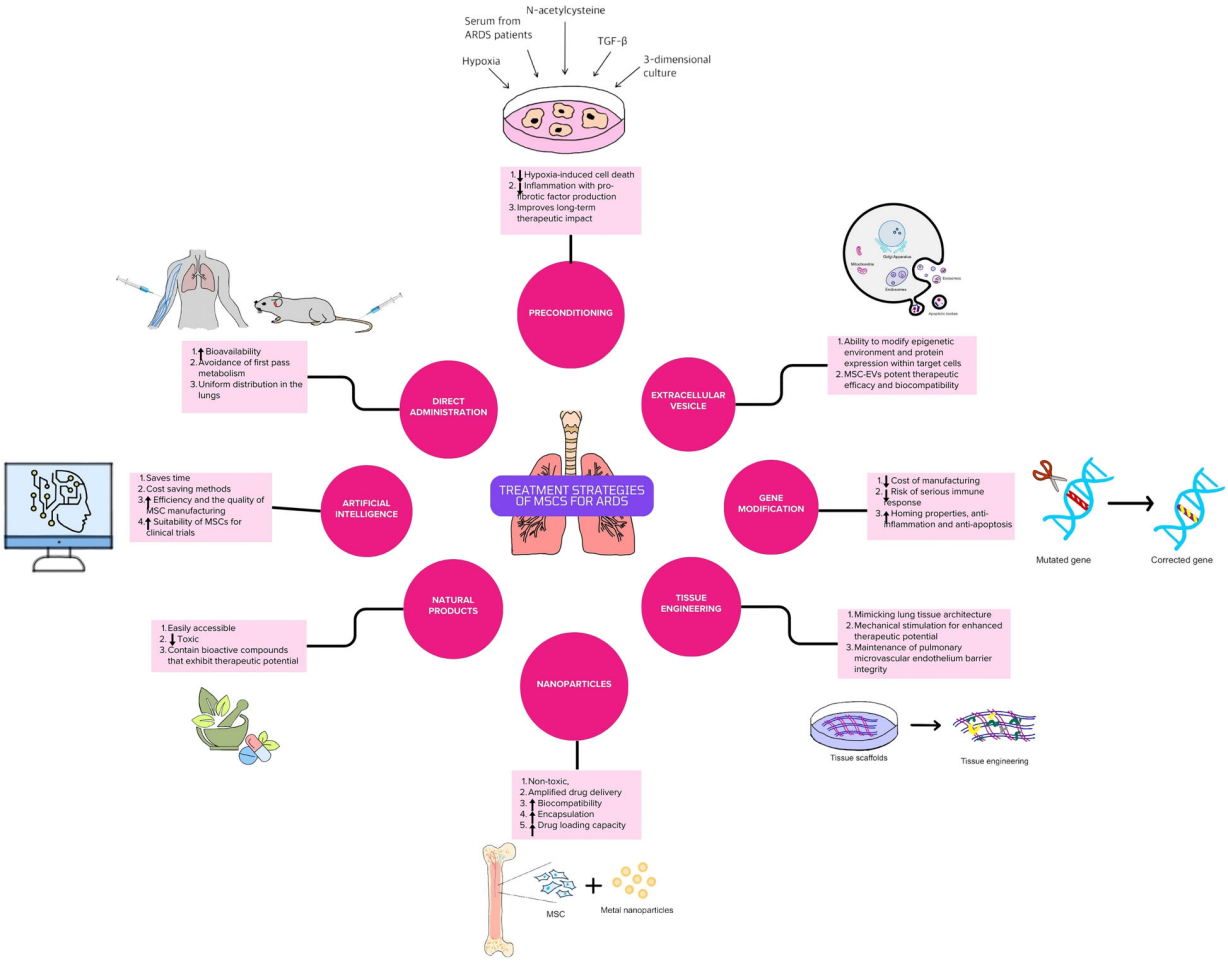
Treatment strategies of mesenchymal stromal/stem cells (MSCs) for acute respiratory distress syndrome (ARDS).

In conclusion, MSC therapy for ARDS has shown promising results in preclinical and clinical studies, but there are still many challenges and limitations that need to be addressed before its widespread application. More research is needed to optimize MSC delivery protocols, standardize MSC quality control, and evaluate the long-term efficacy and safety of MSC therapy for ARDS. Additionally, the underlying mechanisms of MSC therapy need to be further elucidated to improve MSC potency and specificity. The development of approaches, summarized in Figure 2, including direct administration, tissue engineering, EVs, nanoparticles, natural products, AI, gene modification, and preconditioning of MSCs may offer novel and effective treatment options for ARDS, especially for COVID-19 patients, but more rigorous and comprehensive investigation are required to overcome the current barriers in clinical translation.

## Future perspectives

One key future recommendation is the need for large-scale, randomized, and placebo-controlled clinical trials to evaluate the safety and efficacy of MSC-based therapies in ARDS. This will help determine the optimal dosing and delivery routes for MSCs and recognize the potential side effects. The long-term safety and efficacy of MSC-based therapies need to be evaluated, including their potential to cause tumorigenesis or alter the immune response.

The use of gene modification and preconditioning of MSCs may enhance their therapeutic potential and improve their survival and engraftment in the lungs. Gene modification approaches, such as overexpression of anti-inflammatory or pro-regenerative genes, may enhance immunomodulatory and regenerative properties of MSCs in the lungs. Preconditioning of MSCs with hypoxia, cytokines, or other stimuli may also enhance their survival and function in the lung microenvironment. Another recommendation is the development of novel biomaterials and scaffolds that can enhance the survival and function of MSCs in the lung microenvironment. 3D-printed scaffolds and MSC sheets have shown promise in preclinical studies, but further optimization of these approaches is required to enhance their therapeutic potential. Other tissue engineering approaches, such as combining MSCs with extracellular matrix (ECM) components or growth factors, may also enhance their regenerative capacity in the lungs.

EVs derived from MSCs are emerging as a promising therapeutic approach for ARDS due to their ability to mediate intercellular communication and modulate the immune response. Further studies are needed to determine the optimal isolation and purification methods for MSC-derived EVs and their mechanism of action in ARDS. In addition, the use of nanoparticles for targeted delivery of MSC-derived EVs or other therapeutic agents to the lungs is another area that warrants further investigation. Natural products, on the other hand, such as plant-derived compounds or herbal extracts, have widely shown to have immunomodulatory and anti-inflammatory properties and may enhance the therapeutic potential of MSC-based therapies. Further studies are needed to evaluate the efficacy of combining natural products with MSCs for the treatment of ARDS. Finally, AI technology, specifically machine learning approaches, may help optimize the use of MSC-based therapies in ARDS. AI algorithms can be used to predict the optimal dosing and delivery routes for MSCs based on patient-specific data, as well as to identify patients who are most likely to benefit from MSC-based therapies. AI can also be used to analyze large-scale genomic and proteomic datasets to identify potential targets for gene modification or preconditioning of MSCs to enhance their therapeutic potential.
